# How to counteract the lack of donor tissue in cardiac surgery? Initial experiences with a newly established homograft procurement program

**DOI:** 10.1007/s10561-023-10087-z

**Published:** 2023-04-25

**Authors:** Martin O. Schmiady, Ramadan Jashari, Renato Lenherr, Stefan Regenscheit, Dave Hitendu, Martin Wendt, Stefanie Schiess, Martin Schweiger, Michael Hofmann, Juri Sromicki, Andreas Flammer, Markus J. Wilhelm, Robert Cesnjevar, Thierry Carrel, Paul R. Vogt, Carlos A. Mestres

**Affiliations:** 1 Clinic for Cardiac Surgery, University Heart Center, University Hospital Zurich, Rämistrasse 100, CH-8091 Zurich, Switzerland; 2 European Homograft Bank (EHB), University Hospital St. Luc, Brussels, Belgium; 3 Donor Care Association, University Hospital Zurich, Zurich, Switzerland; 4 Department of Congenital Cardiovascular Surgery, University Children’s Hospital Zurich, Zurich, Switzerland; 5 Department of Surgery and Transplantation, Swiss HPB Centre, University Hospital Zurich, Zurich, Switzerland; 6 Clinic for Cardiology, University Heart Centre, University Hospital Zurich, Zurich, Switzerland; 7 Children’s Research Center, University Children’s Hospital Zurich, University Zurich, Zurich, Switzerland

**Keywords:** Homografts, Heart valve allografts, Transplantation, Cardiovascular tissue banking, Organ donation, Tissue donation

## Abstract

Homograft heart valves may have significant advantages and are preferred for the repair of congenital valve malformations, especially in young women of childbearing age, athletes and in patients with active endocarditis. A growing problem, however, is the mismatch between tissue donation and the increasing demand. The aim of this paper is to describe the initiation process of a homograft procurement program to attenuate the shortage of organs. A comprehensive description of the infrastructure and procedural steps required to initiate a cardiac and vascular tissue donation program combined with a prospective follow-up of all homografts explanted at our institution. Between January 2020 and May 2022, 28 hearts and 12 pulmonary bifurcations were harvested at our institution and delivered to the European homograft bank. Twenty-seven valves (19 pulmonary valves, 8 aortic valves) were processed and allocated for implantation. The reasons for discarding a graft were either contamination (*n* = 14), or morphology (*n* = 13) or leaflet damage (*n* = 2). Five homografts (3 PV, 2 AV) have been cryopreserved and stored while awaiting allocation. One pulmonary homograft with a leaflet cut was retrieved by bicuspidization technique and awaits allocation, as a highly requested small diameter graft. The implementation of a tissue donation program in cooperation with a homograft bank can be achieved with reasonable additional efforts at a transplant center with an in-house cardiac surgery department. Challenging situations with a potential risk of tissue injury during procurement include re-operation, harvesting by a non-specialist surgeon and prior central cannulation for mechanical circulatory support.

## Introduction

Valve replacement remains the last surgical option for patients with severe valve dysfunction unsuitable for valve repair. The valvular pathology itself, the valve and patient body size, patient age, and the expected growth in the pediatric population make conduit selection a challenging decision. The limited durability of biological valves is the reason why mechanical valve prostheses are the most frequently used prosthetic type in children and young adults. However, strict postoperative anticoagulation therapy is then mandatory and must be maintained for life. Moreover, none of the industrially manufactured valves have any growth potential. Human homograft valves (allografts) have been used for over six decades. The first implantation of a homograft in the descending aorta was performed in 1956 by Murray in Toronto (Murray 1956). Since then, they have been increasingly used for valve replacement. Their orthotopic use had been established since the description by Ross (1962) and Barratt-Boyes (1965). At that time, they were the only successful biological heart valves aside from mechanical prosthesis. Even if they do not meet all of the properties of a hypothetically ideal heart valve, homografts offer some advantages. They are non-thrombogenic, therefore require no postoperative anticoagulation. They have no opening or closing click noise and restore the anatomy of the aortic or pulmonary outflow tract, which ensures superior hemodynamics. Their resistance to infections make them an ideal graft material in active and destructive infective endocarditis. One of the main disadvantages is their restricted availability, which is dependent on a human donor pool. With a growing number of patients requiring valve replacement, it is clear, that homografts have to be reserved for special situations. While the overall number of cardiac transplants is decreasing, options for tissue donation must be evaluated in every multiorgan donor and implemented in order to increase the availability of human valves. The present work describes how a tissue donation program can be set up and summarizes the initial results.

## Methods

### Study design

This is a retrospective analysis and narrative review of the organization and initial results of the cardiac tissue donation program at University Hospital Zurich, Switzerland.

### Inclusion/exclusion criteria

The donor exclusion criteria were based on age (> 55y for AV, > 70y for PV), clinical and behavioral history (social contact history, contact with toxic substances such as intravenous drug abuse or heavy metals), travel in endemic areas and contact with persons at risk for COVID-19 disease. Table 1 summarizes the current exclusion criteria.

**Table 1:** Exclusion criteria

Age	> 55 years for aortic valve
	> 70 years for pulmonary valve
*Clinical and behavioral history*	
Sexual transmitted disease	HIV 1&2, Hepatitis B&C, HTLV, Syphilis
Donor at risk for AIDS	Intravenous drug abuse, sexual contact with multiple partners, recent imprisonment (last 6 month), exposure to suspicious blood products
Transmissible infectious diseases	Q Fever, Brucellosis, Malaria, Tuberculosis, Creutzfeldt- Jakob dementia, West Nile Virus, COVID-19
	Active infection, septicemia
Malignant disease	Except: basocellular skin epitheliomas and some early cancers (e.g. uterine, cervical, etc.)
Chemotherapy	
Immunosuppression	Donor with altered immune competence or with corticosteroids on a long-term basis (i.e., between 4 years for daily dose of 10 mg and 1 year for a daily dose of 20 mg)
Autoimmune disorders	
Connective tissue disorder	Marfan Syndrome and similar disorders
Unknown cause of death	
*Morphologic characteristics*	
Vascular diseases	Dilatation of Aortic root, ascending Aorta, pulmonary trunk
	Atheroma, severe calcification or other malformations of arterial segments
Congenital and degenerative valve alteration	Bicuspid, monocuspid, quadricuspid
	Malformation of the leaflets (important fusion or fibrosis, large fenestrations)
	Tears or cuts in the leaflets
	Signs of old or active Endocarditis

### Donor selection and indications

To exclude any active viral contamination, the potential donors are systematically tested for COVID-19 (PCR) prior to being accepted for tissue donation. Furthermore, the blood sample is collected prior to or during the heart removal for testing for hepatitis B and C, HIV 1&2, HTLV 1&2 and syphilis. Also, the NAT-HIV, NAT-HBV and NAT-HCV test is mandatory for all tissue donors.

In our institution, patients undergoing heart transplantation gave informed consent for tissue donation (living donors or donor during the “domino procedure”). From the donors undergoing donation after brain death (DBD) or donation after cardiocirculatory determined death (DCD), consent was obtained by consulting the national register or during an interview with the nearest relatives or legal representatives. Indications for DBD/DCD or heart transplantation are summarized in Table 2.

**Table 2:** Indications for Tissue Donation

Indication	Reason	Number of patients
Donation after brain death (DBD) and Donation after cardiocirculatory determined death (DCD)		
	Cardiogenic shock	4
	Hypoxic brain injury	10
	Intracerebral hemorrhage	9
Heart Transplantation		
	Ischemic cardiomyopathy	2
	Dilated cardiomyopathy	2
	Hypertrophic cardiomyopathy	1

### Ethics statement

All procedures performed in this study were in accordance with the ethical standards of the institutional and/or national research committee and with the 1964 Helsinki declaration and its later amendments or comparable ethical standards. This study was approved by the institutional review board of the University Hospital Zurich and the cantonal ethics committee Zurich, Switzerland (ID no. 2022–01,721). Patients or their relatives gave informed consent for tissue donation and the use of medical data. Patients who had rejected organ or tissue donation and cases in which no information about the presumed willingness of the patient was available were excluded from donation and from the study.

### Operative procurement technique

Cardiac tissue procurement was carried out after verification of the status of donor consent, obtained either directly from the donor themself (living donor), consulting the national registry or after discussion with the nearest relatives of the donor (after death). At the time of the study Switzerland had adopted the “opt-in” system, meaning that citizens need to explicitly express their consent to be a donor (Schaub et al. 2019). Cardiac tissue harvesting was carried out in the operating room with a controlled environment and laminar flow including air filtration using HEPA-filters. Hearts were procured by either a cardiac or thoracic surgeon. All hearts were procured through a median sternotomy, which gives excellent visualization and access to the entire heart and the great thoracic vessels. Optimal access is key to success as it allows the surgeon to cut the aortic and pulmonary conduits as distal as possible.

After median sternotomy, the pericardium was opened in a Y-shaped manner. Pericardial stay sutures were placed. The heart was removed by transection of the inferior vena cava and the left atrium. In case of simultaneously lung procurement, care must be taken to get a sufficient length of the pulmonary trunk above the pulmonary valve (at least 2–3 cm). In patients in which the lungs are not donated, the pulmonary veins can be transected at the level of their connection with the left atrium. Next the main pulmonary artery before the bifurcation (in case of lung procurement) or the pulmonary branches as high as possible are transected. The pulmonary arterial branches are important for production of the non-valved pulmonary conduits.

**Fig. 1:**
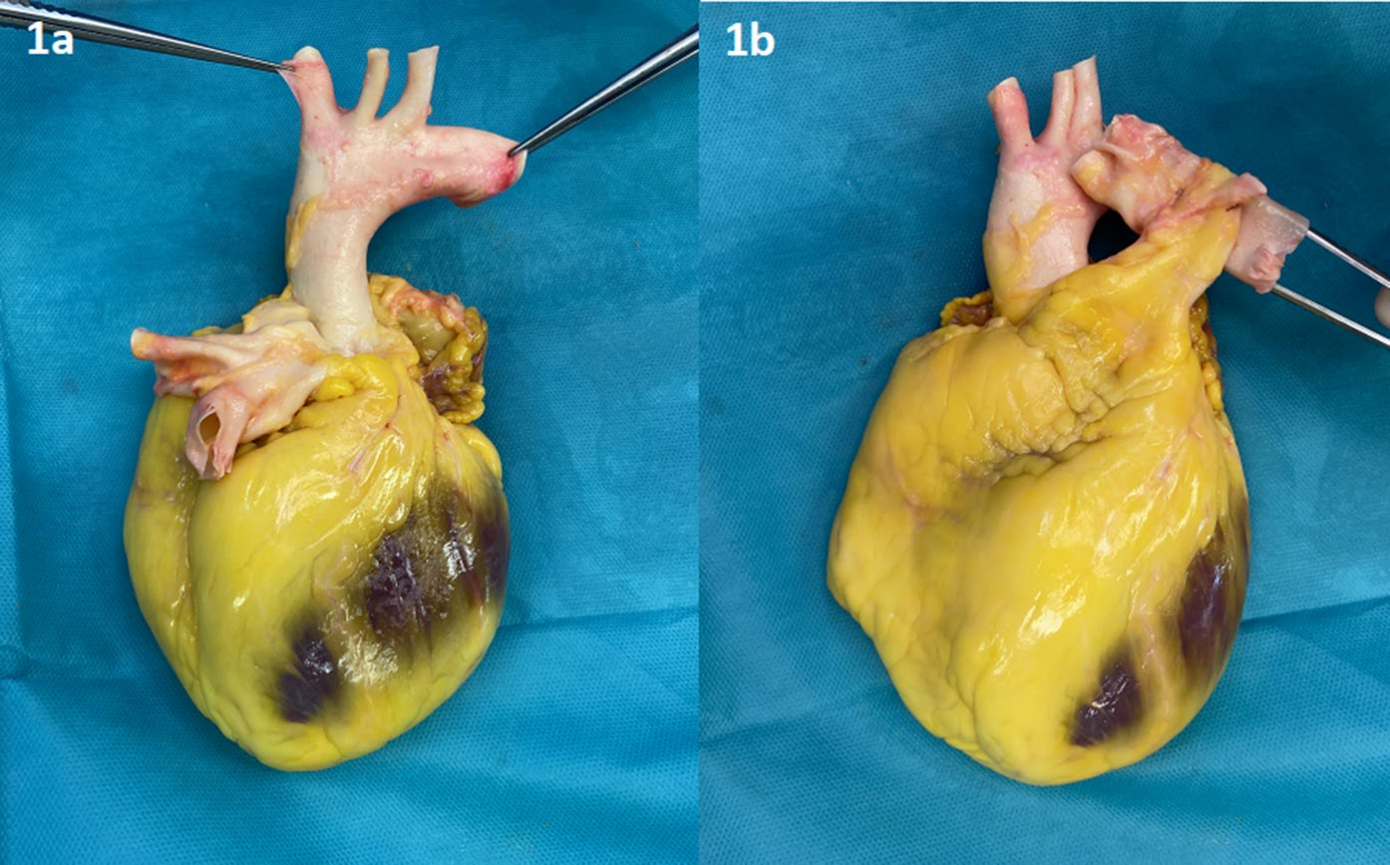
1a and 1b. Transected heart with aortic arch (1a) and pulmonary trunk (1b)

At the end, the superior caval vein and the distal arch (behind the left subclavian artery) are transected (Fig. 1 1a and 1b). The heart is then rinsed with saline, in order to remove remaining blood and avoid clot formations, which could be an ideal environment for infections.

### Shipping

The procured heart is packed in a sterile manner in a plastic container, filled with 500 ml of cold, sterile saline at around + 4 °C and shipped in a thermal box to the European homograft bank (EHB). During heart procurement, blood samples are obtained for later quality control analyses in the EHB. All shipping materials and the kit of blood sampling have been prepared by the EHB and sent in advance to the University Hospital Zurich. The EHB responsible person organized the transport of the procured heart to EHB, as the procured tissues must be delivered within 24 h from the moment of cardiac arrest [Jashari 2010].

### Processing and cryopreservation

For the optimal evaluation of donated cardiac tissues, a good manufacturing practice facility for tissue processing is available at EHB. The facility is compliant with the EU recommendations, regulations, and quality standards for cardiovascular tissue banking (Jashari et al. 2004; Practices 2023). Allograft dissection and preparation are performed in a Class A vertical laminar flow, in 2 separate work stations. During preparation, the arterial trunks (aortic root and pulmonary artery) are separated and surrounding fat tissues removed in order to allow penetration of the antibiotic solution during the incubation in an antibiotic cocktail. Morphological evaluation of the valve leaflets and a functional test were thereafter performed. Finally, measurement of the valve diameter and length of the arterial conduit was carried out. After collection of samples for bacteriological testing the grafts are incubated in the antibiotic cocktail for 20 to 48 h at + 4 °C. This is followed by another microbial test. Subsequently, the allografts were cryopreserved. The storage of heart valve allografts was possible using liquid nitrogen vapors in extreme low temperature conditions (below − 130 °C) after control rate cryopreservation in a Planer 560/16 system (Planer Ltd., Middlesex, United Kingdom), with a computer-guided program.

### Tissue follow-up

All 28 hearts that had been sent to the EHB within the initial experience were followed up actively. Microbiological tests and morphological abnormalities were documented as were any reasons for destruction. The fate of each graft was followed from explantation through processing and quality control to storage or implantation.

## Results

Between January 2020 and May 2022, 28 organ donors were included in this study. The mean patient age was 55.4 years, and 54% were male. Patients characteristics are represented in Table 3. Twenty-three hearts were obtained from DBD and DCD donors during a multiorgan procurement. Five hearts were explanted from heart transplant recipients (RHT donors). Reason for heart transplantation in this group was ischemic cardiomyopathy in 2, dilated cardiomyopathy in 2 and hypertrophic cardiomyopathy in 1 patient. In 10 of the DBD/DCD patients the lungs were accepted for transplantation and were removed during the same procedure. In these cases, the pulmonary bifurcation was left attached to the lung package and was not available for tissue donation.

**Table 3:** Patient demographics

Patient characteristics	
Gender male n (%)	15 (54)
Mean age (years)	55.4 (11.4–69.8)
Mean weight (kg)	75 ( 30–120)
Mean hight (cm)	170 (104–185)
BMI	25.4 (14.3–77.4)
BSA (m^2^)	1.89 (1.12–2.42)

**Fig. 2:**
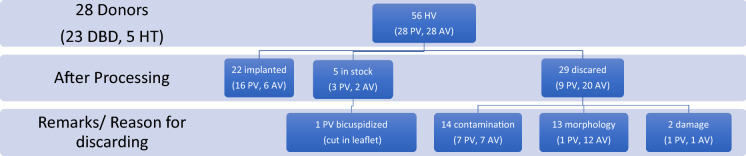
Donated organs and heart valves

**Fig. 3:**
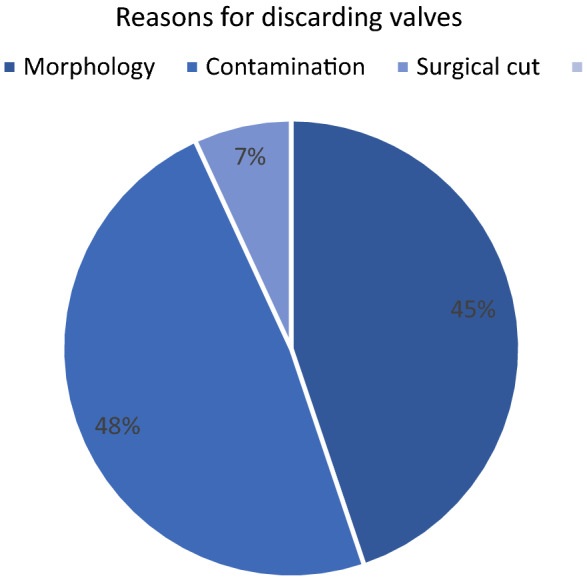
Reasons for discarding valves (51% discarded)

A total of 28 hearts and 12 pulmonary bifurcations/ non-valved pulmonary conduits could be harvested in this cohort. Twenty-six organs were harvested by an experienced cardiac surgeon. Two hearts were removed by a thoracic surgeon during simultaneous lung procurement. Figure 2 gives an overview of the donated organs and procured heart valves and describes their fate. All together 56 heart valves (28PV and 28AV) were sent to the EHB. 27 heart valves (19 pulmonary valves, 8 aortic valves) were processed and allocated for implantation. Figure 3 illustrates the reasons for discarding a heart valve allograft. Reasons included contamination (*n* = 14), morphology (*n* = 13) and leaflet damage (*n* = 2). Five homografts (3 PV, 2 AV) were cryopreserved and are in storage awaiting allocation by the EHB. At the time of writing the manuscript, 22 grafts (16 PV, 6 AV) were allocated and successfully implanted in different centers in Europe. One pulmonary homograft with a leaflet cut was salvaged by bicuspidization technique and awaits allocation, and it is a highly sought after small diameter graft. In addition to the heart valves mentioned, 12 non-valved pulmonary conduits were also obtained and 6 had been implanted at the time this article was written. Three conduits are still in stock and awaiting allocation and 3 had to be discarded due to contamination (1 Candida albicans, 1 Propionibacterium acnes, 1 Saccharomyces).

## Discussion

The ideal valve substitute has not yet been replicated, but there are presently multiple options and novel experimental approaches/devices with each of them presenting some subset of the desirable characteristics (Kalfa 2022).

One widely used option is human homograft heart valves. Currently, the homograft valves undergo cryopreservation and are stored in the liquid nitrogen vapour. These grafts have shown excellent hemodynamics with low thrombogenicity, and they have favorable operative handling characteristics and achieve good intraoperative hemostasis. Homografts have a low risk of endocarditis and may be favorable in acute/or destructive infective endocarditis (Musci et al. 2010). To improve long-term durability, decellularization technologies have been proposed and successfully implemented in everyday clinical practice (Simon, et al. 1006). The prospective European multicenter ESPOIR trial showed significantly better freedom from explant and less structural valve degeneration at 5 years follow up for decellularized pulmonary homografts when compared to it competitors (Bobylev et al. 2022). Similar promising results have been shown by the prospective, multicenter European trial on decellularized allografts for aortic valve replacement (Horke et al. 2020a, b). However, all of these technologies are based on a sufficiently large donor pool to provide the human heart valves required for subsequent processing.

**Fig. 4:**
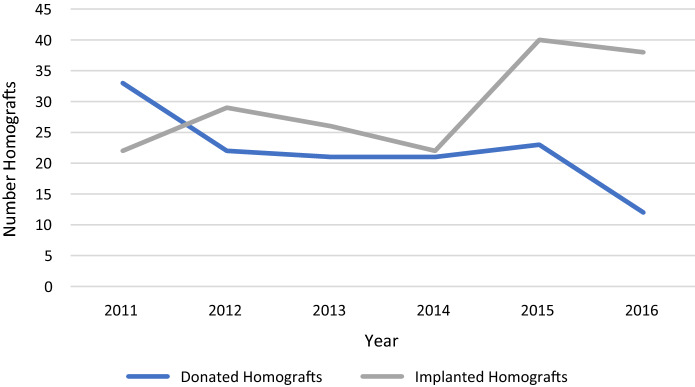
Mismatch between donated and implanted homografts

To overcome the current shortage, emergency solutions, such as bicuspidized pulmonary homografts, were introduced and have shown comparable intermediate-term durability (Michler et al. 1994; Shih et al. 2010). However, these approaches do not change the overall availability of homografts. Therefore, it makes clearly sense to look for other solutions at the very beginning of the supply chain. A central point is to increase awareness in the population of the need for organ donation and to establish an appropriate infrastructure for effective donation of the hearts for valve allografts. Comparable to the global trend, a decline in cardiac tissue donations has been observed in Switzerland, while the need was simultaneously increasing (BAG 2022). From 2011 to 2016, donations decreased by around 64% while the requirement increased from 22 to 38 homografts, which had to be imported. This mismatch creates an imbalance between supply and demand (Fig. 4) which was the main driver to start our own homograft donation program.

### The need for specialization

In the present study, 26 hearts were harvested by an experienced cardiac surgeon. Two hearts were removed by a non-specialist surgeon during simultaneous lung procurement. Unfortunately, both hearts were excised very close to the commissures of the pulmonary valve resulting in leaflet damage. One of these grafts had to be discarded and was therefore not longer available for allocation. The other pulmonary homograft with a leaflet cut was salvaged by bicuspidization technique and awaits allocation. This experience speaks in favor of organ removal through a cardiac surgeon who may posess more precise cardiac anatomical knowledge and experience. Especially in DCD donors the heart and vascular structures are bloodless and collapsed, complicating anatomical identification and retrieval. Thus, we highly recommend that a cardiac surgeon always be involved in the procurement of a heart.

### Challenging procurement scenarios

Two further situations may be challenging: a redo case and patients with prior or still extracorporeal circulatory support in situ. In re-do situations it is common to find scar tissue and adhesions, which may challenge the overview and preparation. In this setting tissue damage may occur more easily. Therefore, these patients may not be the ideal candidates for cardiac tissue donation and should be evaluated by experienced procurement teams. In experienced hands however, these hearts may also be used for tissue donation. In children and young adults most of the heart valve allografts are used for reconstruction of the right ventricular outflow tract (RVOT). For this reason, there is mainly a lack of pulmonary homografts in this population. Therefore, it makes sense to evaluate as well patients after aortic valve replacement or valve sparing procedures and take at least the pulmonary valve, when untouched previously. Patients on mechanical circulatory support may also make organ procurement more difficult. Especially in the cases of central cannulation, the cannulation side may be close to the commissural level of the heart valves. Thus, an organ removal with enough distance to the valve can be challenging. Our study includes one patient on veno-arterial extracorporeal membrane oxygenation support (V-A ECMO) with central cannulation. In this patient, the aortic valve was damaged during procurement due to insufficient distance between cannulation side and leaflet commissures. The damaged valve had to be discarded and was therefore not available for further use as homograft.

In this study, 5 of the hearts were harvested from recipients of a heart transplantation. In these scenarios, proper operative planning with the surgeon performing the transplantation is essential. In most of the cases the recipient heart can be transected with sufficient distance to the heart valves. However, our experience has shown that in the case of a heart transplantation, the surgeon performing the transplantation tends to remove as little recipient vascular tissue as possible. This may be driven by concerns about not having enough tissue available with consecutive tension on the arterial anastomoses. In the case of normal anatomy, these concerns are unfounded, as the donor heart has sufficient artery length for a safe organ implantation.

### Patient specific factors

**Fig. 5:**
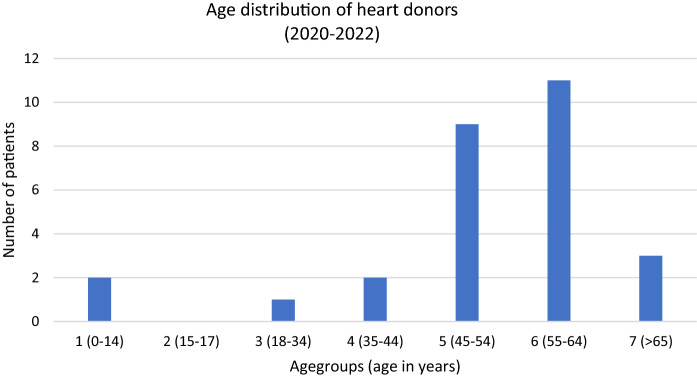
Age distribution of heart donors

Figure 5 the main driver to start donated hearts. With only 3 donors in the age group < 35 years, our study confirms the observed lack of small grafts. An important next step is to focus on this cohort and evaluate ways to increase the pool of pediatric donors. An advantage over organ donation is that the cardiac tissue, used for homograft processing, can be removed hours after the patient's death. In the event of the death of a young patient, this gives the parents time to say goodbye and mourn at the bedside. Tissue removal can then be carried out after a few hours in a sterile environment (maximum 6 h if the donor body was not refrigerated; maximum 24 h if the donor body was refrigerated). This method could improve the acceptance of tissue donation, but requires intensive education of patients, relatives and medical staff. Another focus should be on the donation of the large thoracic vessels. In particular, the pulmonary artery bifurcation that can be used for the reconstruction of the aortic arch in congenital malformations and the descending aorta that may be used in case of infected aortic aneurysm situations. In our study 12 non-valved pulmonary conduits could be obtained in addition to the previously heart valves. As the pulmonary artery bifurcation can be divided in the middle (if sufficient length of the proper pulmonary artery was recovered) it may be used to treat 2 patients.

In the best-case scenario, tissue for 4 recipients can be obtained from a single donated heart. However, looking at Fig. 3, morphological reasons such as atheromas, fat deposits, calcification, unfavorable fenestrations and inefficient decontamination with the antibiotic cocktails (valves 52%, non valved pulmonary arterial conduits 25%) were significant reasons to discarding tissues. Also, grafts from older donors may show advanced degenerative changes. The aortic valve, located in the high-pressure system, usually shows more morphological changes than the pulmonary valve. Accordingly, more aortic valves had to be discarded due to morphological changes. In our study 13 (45%) valves had to be excluded due to morphological reasons, and 12 (92%) of these valves were aortic valve grafts. In the future, the number of discarded tissues may even increase more, because of the advanced age of donors. Therefore, renewed efforts are needed to increase the pool of donors, seeking to overcome the allograft shortage that has already been observed.

## Limitations

This is a retrospective observational study, from a single center, with relatively small numbers of patients. Patients were not randomized or propensity matched to a comparison group. Selection bias of variables may not be excluded.

## Conclusion

This work describes the initial steps and preliminary results with a new organ donation program for cardiac tissues. The essential processes and common pitfalls that need to be avoided are described in a structured manner. Especially in transplant centers, the required infrastructure is available and this needs to be activated and coordinated. The involvement of all disciplines that care for the patient is critical in order to identify possible tissue donors at an early stage. Explanation to the donor's relatives about the final aim of the donated body material is fundamental in order to achieve acceptance for the topic of organ and tissue donation in the society. Clear communication with the different organ harvesting teams in the operating room and the supervision by a cardiac surgeon are basic requirements for safe tissue harvesting.

## Abbreviations

AV: Aortic valve
ARISE: Aortic valve replacement using individualized regenerative allografts
BMI: Body mass index
BSA: Body surface area
DBD: Donation after brain death
DCD: Donation after circulatory death
ECMO: Extracorporeal membrane oxygenation
EHB: European homograft bank
ESPOIR: A European study on decellularized homografts for pulmonary valve replacement
EU: European Union
HEPA: High-Efficiency Particulate Air
HIV: Human immunodeficiency virus
HTLV: Human T-cell leukemia virus
HV: Heart valve
PV: Pulmonary valve
RVOT: Right ventricular outflow tract
V-A: Veno-arterial
Y: Years
